# Supplementary material for: Disease burden and seasonal impact of improving rotavirus vaccine coverage in the United States: A modeling study
Source: PLoS One. 2020 Feb 14;15(2):e0228942. doi: 10.1371/journal.pone.0228942 (PMC7021296; doi:10.1371/journal.pone.0228942)
Supplement: S1 Appendix — S1 Table. Rate of severe rotavirus cases (per 10,000) and percent of severe cases averted for each rotavirus vaccination scenario in 2000 to 2030 assuming random mixing patterns between children visiting pediatricians and family practitioners. S2 Table. Children 0–11 months old percent of severe rotavirus cases averted in post-vaccine era and new vaccine scenario after 2018 in pediatrician and family practitioner populations assuming random mixing patterns between children visiting pediatricians and family practitioners. S3 Table. Rate of severe rotavirus cases (per 10,000) and percent of severe cases averted for each rotavirus vaccination scenario in 2000 to 2030 with assortative mixing patterns assuming 80% of contacts occur within a group and 20% of contacts occur between groups. (DOCX) [file pone.0228942.s001.docx]

## S1 Appendix:

Our model is a system of ordinary differential equations, the full model equations are:

$$\frac{{dM}_{i}}{dt}= {\mu N-eM_{l}-\delta M_{l}+\delta M_{\left( l-1 \right)}+(1-\rho\chi)M}_{(l-1)}-{\mu M}_{l}$$

$$\frac{{dS}_{1i}}{dt}= {eM_{l}-\lambda_{i}S_{1i}-\delta S}_{1i}+{\delta S}_{1\left( i-1 \right)}+(1-\rho\chi)S_{1(i-1)}+\omega R_{i}-\mu S_{1i}$$

$$\frac{{dS}_{2i}}{dt}= \varepsilon_{1}\gamma I_{2i}-\lambda_{i}S_{2i}-{\delta S}_{2i}+{\delta S}_{2\left( i-1 \right)}+\left( 1-\rho\chi\right)S_{2\left( i-1 \right)}+\rho\chi\varepsilon_{1}M_{\left( l-1 \right)}+\rho\chi{\varepsilon_{1}S}_{1\left( i-1 \right)}-\mu S_{2i}$$

$$\frac{{dS}_{3i}}{dt}= \varepsilon_{2}\gamma I_{3i}-\lambda_{i}S_{3i}-{\delta S}_{3i}+{\delta S}_{3\left( i-1 \right)}+(1-\rho\chi)S_{3(i-1)}+\rho\chi\varepsilon_{1}S_{2\left( i-1 \right)}-\mu S_{3i}$$

$$\frac{{dS}_{4i}}{dt}= \varepsilon_{3}\gamma I_{4i}-\lambda_{i}S_{4i}-{\delta S}_{4i}+{\delta S}_{4(i-1)}+(1-\rho\chi)S_{4(i-1)}+\rho\chi\varepsilon_{1}S_{3\left( i-1 \right)}-\mu S_{4i}$$

$$\frac{dI_{ni}}{dt}=\lambda_{i}S_{ni}-\gamma I_{ni}-{\delta I}_{ni}+{\delta I}_{n\left( i-1 \right)}-\mu I_{ni}$$

where $n=1\ldots4$

$\frac{dR_{i}}{dt}=\left( 1-\varepsilon_{q} \right)\gamma I_{ni}+\gamma I_{4i}+\left( 1-\varepsilon_{1} \right)\rho\chi M_{\left( l-1 \right)}+\left( 1-\varepsilon_{1} \right)\rho\chi S_{n\left( i-1 \right)}-\omega R_{i}- \delta R_{i}+\delta R_{\left( i-1 \right)}-\mu R_{i}$ where $n=1\ldots4$

where:

*l* = 1 … 3 represent age groups 0 – 1 month, 2 – 3 months, and 4 – 11 months, respectively.

*i* = 1 … 6 represent age groups 0 – 1 month, 2 – 3 months, 4 – 11 months, 1 – 4 years, 5 – 24 years, and above 25 years, respectively.

*S_ni_* = susceptible to nth rotavirus infection (*n* =1…4) in age group *i*

*I_ni_* =infected by nth rotavirus infection (*n* = 1…4) in age group *i*

*R_i_* = recovered and immune to rotavirus infection in age group *i*

*µ =* rate of birth and mortality

$e$ = rate of loss of maternal immunity

$\delta$ = rate at which individuals in age group *i* age into age group (*i* +1)

$\rho$ = proportion vaccinated

$\chi$ = vaccine efficacy

$\varepsilon_{n}$= risk of becoming re-susceptible after nth rotavirus infections

$\gamma$ = rate of loss of infection

$\omega$= rate of loss of immunity

$\lambda_{i}$= force of infection; rate at which susceptible individuals become infected in age group *i*

Susceptible individuals are infected at a rate (λ(t)) and enter the infectious compartment. Infected individuals either recover from infection at rate $(\gamma)$ and/or become susceptible to subsequent infections. Immunity wanes at rate $(\omega)$ and individuals become susceptible again. This model assumes individuals can have up to four rotavirus infections with decreasing probabilities of infection, disease and severe disease ($\varepsilon$ _1-3_) given the number of previous infections [14]. Individuals are vaccinated with fraction ($\rho$) and vaccine efficacy ($\chi$) and enter the recovery compartment. Individuals who are vaccinated with unsuccessful immunity become susceptible with probability ($\varepsilon$ _1_) to subsequent infections.

The force of infection was calculated as:

$$\lambda_{j}(t)=\beta_{\left( t \right)}c_{j}\sum_{j=1}^{4} s_{jk}\left( \frac{I_{1j}+\sum_{n=2}^{4} rI_{nj}}{N_{j}} \right)$$

where:

*j* = *k* = 1 … 4 represent contact patterns in age group 0 – 1 year, 1 – 4 years, 5 – 25 years, and above 25 years, respectively.

$c_{j}$= count of total contacts in each age group

$s_{jk}$=proportion of contact between group *j* and *k* (*j*=1…4, *k*=1...4)

$I_{nj}$= infected by nth rotavirus infection (*n* = 1…4) in contact patterns *j*

$N_{j}$=total population in contact pattern *j*

*r* = relative infectiousness of non-primary infections

We modeled seasonal variation of rotavirus transmission $\beta(t)$:

$$\beta(t)=q_{l}(1+A cos(2\pi t + ϴ))$$

where $q_{l}$ represents probability of transmission per contact from children in age group *l*, $A$is the amplitude of the seasonal fluctuation and $ϴ$ is the phase angle in years $(t)$.

*l* = 1…4 represent age group <1 year, 1 – 4 years, 5 – 24 years, > 25 years, respectively.

**S1 Table.** Rate of severe rotavirus cases (per 10,000) and percent of severe cases averted for each rotavirus vaccination scenario in 2000 to 2030 assuming random mixing patterns between children visiting pediatricians and family practitioners.

| Year | Status Quo ^a^ | | | Improved FP ^b^ | | | Improved FP+PE ^c^ | | |
| --- | --- | --- | --- | --- | --- | --- | --- | --- | --- |
|  | | Rate | Rate | | Percent Averted compared to 2000-2006 | Percent Averted compared to Status Quo | Rate | Percent Averted compared to 2000-2006 | Percent Averted compared to Status Quo |
| 2000 – 2006 average | | 327 | 327 | |  |  | 327 |  |  |
| 2007 | | 50 | 50 | | 85% |  | 50 | 85% |  |
| 2008 | | 40 | 40 | | 88% |  | 40 | 88% |  |
| 2009 | | 100 | 100 | | 69% |  | 100 | 69% |  |
| 2010 | | 65 | 65 | | 80% |  | 65 | 80% |  |
| 2011 | | 82 | 82 | | 75% |  | 82 | 75% |  |
| 2012 | | 62 | 62 | | 81% |  | 62 | 81% |  |
| 2013 | | 80 | 80 | | 75% |  | 80 | 75% |  |
| 2014 | | 65 | 65 | | 80% |  | 65 | 80% |  |
| 2015 | | 79 | 79 | | 76% |  | 79 | 76% |  |
| 2016 | | 67 | 67 | | 80% |  | 67 | 80% |  |
| 2017 | | 79 | 79 | | 76% |  | 79 | 76% |  |
| 2018 | | 68 | 56 | | 83% | 17% | 46 | 86% | 32% |
| 2019 | | 78 | 58 | | 82% | 26% | 25 | 92% | 68% |
| 2020 | | 69 | 55 | | 83% | 20% | 33 | 90% | 51% |
| 2021 | | 78 | 57 | | 82% | 26% | 36 | 89% | 54% |
| 2022 | | 70 | 55 | | 83% | 21% | 30 | 91% | 56% |
| 2023 | | 77 | 57 | | 83% | 26% | 31 | 90% | 60% |
| 2024 | | 70 | 56 | | 83% | 21% | 32 | 90% | 54% |
| 2025 | | 77 | 57 | | 83% | 26% | 32 | 90% | 58% |
| 2026 | | 71 | 56 | | 83% | 21% | 32 | 90% | 54% |
| 2027 | | 77 | 57 | | 83% | 26% | 32 | 90% | 57% |
| 2028 | | 71 | 56 | | 83% | 21% | 33 | 90% | 54% |
| 2029 | | 77 | 57 | | 83% | 26% | 33 | 90% | 57% |
| 2030 | | 72 | 57 | | 83% | 21% | 33 | 90% | 54% |

1. 85% vaccination coverage for children visiting pediatricians and 45% for children visiting family practitioners (total 78.6% current vaccination coverage).
2. 85% vaccination coverage for children visiting pediatricians and family practitioners.
3. 95% vaccination coverage for children visiting pediatricians and family practitioners.

**S2 Table.** Children 0 – 11 months old percent of severe rotavirus cases averted in post-vaccine era and new vaccine scenario after 2018 in pediatrician and family practitioner populations assuming random mixing patterns between children visiting pediatricians and family practitioners.

|  | **Status Quo ^a^ Rate^c^** | | **Improved FP ^b^ Rate^c^** | | |
| --- | --- | --- | --- | --- | --- |
| **Time period** | **Pediatricians** | **Family practitioners** | | **Pediatricians** | **Family practitioners** |
| 2000-2006 | 993 | 993 | 993 | | 993 |
| 2010-2017 | 143 | 176 | 143 | | 176 |
| 2018-2029 | 148 | 182 | 114 | | 115 |
| **2018 – 2029 average percent of averted severe rotavirus cases compared to Status Quo** | -- | -- | 23% | | 37% |
| **2018 – 2029 average percentage averted severe rotavirus cases compared to pre-vaccine era** | 85% | 82% | 89% | | 88% |
| **Additional percentage averted from improved vaccine coverage in Improved FP** | -- | -- | 4% | | -- |

1. 85% vaccination coverage for children visiting pediatricians and 45% for children visiting family practitioners (total 78.6% current vaccination coverage total 78.6%).
2. 85% vaccination coverage for children visiting pediatricians and family practitioners (0% improved vaccine coverage in pediatrician population, 45% improved vaccine coverage in family practitioner population).

c. Rate of severe RVGE per 10,000 population

**S3 Table.** Rate of severe rotavirus cases (per 10,000) and percent of severe cases averted for each rotavirus vaccination scenario in 2000 to 2030 with assortative mixing patterns assuming 80% of contacts occur within a group and 20% of contacts occur between groups.

| Year | Status Quo ^a^ | | | Improved FP ^b^ | | Improved FP+PE ^c^ | | |
| --- | --- | --- | --- | --- | --- | --- | --- | --- |
|  | | Rate | Rate | Percent Averted compared to 2000-2006 | Percent Averted compared to Status Quo | Rate | Percent Averted compared to 2000-2006 | Percent Averted compared to Status Quo |
| 2000 – 2006 average | | 326 | 326 |  |  | 326 |  |  |
| 2007 | | 51 | 51 | 84% |  | 51 | 84% |  |
| 2008 | | 68 | 68 | 78% |  | 68 | 78% |  |
| 2009 | | 91 | 91 | 73% |  | 91 | 73% |  |
| 2010 | | 65 | 65 | 77% |  | 65 | 77% |  |
| 2011 | | 83 | 83 | 75% |  | 83 | 75% |  |
| 2012 | | 65 | 65 | 78% |  | 65 | 78% |  |
| 2013 | | 81 | 81 | 75% |  | 81 | 75% |  |
| 2014 | | 68 | 68 | 77% |  | 68 | 77% |  |
| 2015 | | 79 | 79 | 76% |  | 79 | 76% |  |
| 2016 | | 70 | 70 | 77% |  | 70 | 77% |  |
| 2017 | | 79 | 79 | 76% |  | 79 | 76% |  |
| 2018 | | 71 | 53 | 82% | 23% | 46 | 84% | 33% |
| 2019 | | 78 | 45 | 86% | 44% | 15 | 96% | 84% |
| 2020 | | 72 | 60 | 81% | 18% | 27 | 91% | 62% |
| 2021 | | 78 | 51 | 84% | 33% | 40 | 86% | 44% |
| 2022 | | 73 | 55 | 82% | 24% | 28 | 91% | 63% |
| 2023 | | 78 | 53 | 83% | 32% | 27 | 91% | 62% |
| 2024 | | 73 | 54 | 82% | 25% | 30 | 90% | 57% |
| 2025 | | 77 | 54 | 83% | 30% | 31 | 90% | 59% |
| 2026 | | 74 | 54 | 82% | 26% | 30 | 90% | 59% |
| 2027 | | 77 | 54 | 83% | 29% | 30 | 90% | 58% |
| 2028 | | 74 | 54 | 82% | 27% | 31 | 90% | 58% |
| 2029 | | 77 | 54 | 83% | 28% | 31 | 90% | 58% |
| 2030 | | 75 | 54 | 83% | 27% | 31 | 90% | 58% |

1. 85% vaccination coverage for children visiting pediatricians and 45% for children visiting family practitioners (total 78.6% current vaccination coverage).
2. 85% vaccination coverage for children visiting pediatricians and family practitioners.
3. 95%vacccination coverage for children visiting pediatricians and family practitioners.
