# Supplementary material for: Disease burden and seasonal impact of improving rotavirus vaccine coverage in the United States: A modeling study
Source: PLoS One. 2020 Feb 14;15(2):e0228942. doi: 10.1371/journal.pone.0228942 (PMC7021296; doi:10.1371/journal.pone.0228942)
Supplement: Attachment — Submitted filename: Response to Reviewers_ChinEn Ai.docx [file pone.0228942.s002.docx]

**Response to Reviewers**

**Comments from the reviewers:**

**Reviewer #1:** In this paper, Ai et al use a standard mathematical model to investigate the impact of improving coverage with rotavirus vaccine on rotavirus disease burden, and the effect it could have on the biennial patterns that have emerged post vaccine introduction. They test increasing coverage in two patient populations: those attended by pediatricians and those attended by family practitioners. They project significant reductions in disease burden (compared to current levels) when coverage is increased among patients of family practitioners alone, and larger reductions when coverage is increased among patients of both family practitioners and pediatricians. With increased coverage, they note a disappearance of the biennial pattern of rotavirus incidence (it is replaced by a reduced annual pattern), supporting the hypothesis that biennial patterns arise from an inter-year accumulation of susceptible persons in settings with moderate coverage. This is a well written paper, that is concise and understandable even for non-modelers, with clear conclusions that are directly derived from their results, and with an important public health message. The model structure has been built to emulate natural rotavirus disease and transmission. I only have a few minor comments.

**Response**: Thank you for your comments.

Introduction:
Line 60: Typographical error, it should say “$1 billion in direct and indirect costs to the U.S.”

**Response**: The typographical error is corrected

**Edits to text (line 60)**: “$1 billion in direct and indirect costs to the U.S.”

Line 63: Typographical error, it should say “rotavirus incidence has declined between 57%” (erase ‘in the’)

**Response**: The typographical error is corrected

**Edits to text (line 63)**: ” that rotavirus gastroenteritis (RVGE) incidence has declined between 57% - 89%”

Methods:
Line 110 and Table 1: The text mentions the model is age-structured with 6 age-groups, yet the table shows parameters only for 4 age-groups, would clarify. Perhaps estimated transmission parameters are reported as larger age-groups.

**Response**: Indeed, age groups 0 – 1 months, 2 – 3 months, and 4 – 11 months in the model shared the same group of estimated age specific transmission parameters: age-group <1 year.

**Edits to text (line 143 – 144)**:” We estimated four age specific transmission parameters ($q_{1}$ for <1 year, $q_{2}$ for 1-4 years, $q_{3}$ for 5-24 years, and $q_{4}$ for > 25 years)”

Line 145: Would expand on, explain more, how the calibration of vaccine effectiveness allows the model to capture the biennial patterns.

**Response**: A likely cause of biennial patterns of disease in the U.S. is the accumulation of susceptibles that results from modest vaccine coverage. Vaccine efficacy and the rate of loss of immunity both impact the accumulation of susceptibles over time so calibrating these parameters allows the model to capture the biennial patterns that are observed in the U.S.

We add the explanation of the factors driving biennial patterns to the introduction and methods to explain why we calibrate vaccine effectiveness and rate of loss immunity to capture biennial patterns. Edits to text (lines 83 – 85): “In general, biennial patterns of RVGE could be induced by factors that govern the rate of new susceptibles. These include incomplete vaccine coverage, imperfect vaccine efficacy, and birth rate (Pitzer 2009; Pitzer 2011, Shah 2018).

**Edits to text (156 – 164)**: “Biennial epidemic patterns in the U.S. may be driven by a slower accumulation of susceptibles following modest vaccine coverage. By calibrating the vaccine efficacy and rate of loss of immunity parameters (i.e., increasing vaccine efficacy and lowering the rate of loss of immunity) we can slow the accumulation of the simulated susceptible population which allows the model to capture biennial epidemic patterns.”

Discussion:
Line 278: The line “partial immunity when individuals are susceptible” might need to be rephrased, this is not clear.

**Response**: In lines 286 – 288, partial immunity (i.e. subsequent infection can still occur but at reduced rate) was explained

**Edits to text (lines 288 – 291)**: Another modeling study that predicted high vaccine coverage with biennial patterns assumed one-year duration of complete immunity after previous infection, then, individuals are susceptible to infection again with partial immunity

Line 311: The public health relevance of the additional 4 percent in reductions in severe rotavirus gastroenteritis when assuming assortative transmission by practitioner is unknown, and authors might want to acknowledge this, or translate to absolute numbers.

**Response**: Our raw data shows that an additional 7000 RVGE cases are averted when assuming assortative mixing patterns compared to random mixing patterns in Improved FP.

**Edits to text (lines 233 -235)**: Since there is no improved vaccine coverage in the PE population in Improved FP, this additional 4% of severe cases (7000 cases lower) averted in PE population are indirect benefits of improved vaccine coverage from FP population.

Line 320: If the intensity in the year to year variability of the biennial patterns were not captured, then the authors might want to acknowledge that other factors might be at play, or hypothesize which ones.

**Response**: We rephrased our statement: Our model did capture the intensity in the year to year variability in the present biennial epidemic patterns, however, the actual RVGE cases in even year rotavirus season were much lower than the value predicted by our model (Aliabadi 2015; Getachew 2018).

**Edits to text (lines 333 – 336)**: Second, relatedly, our model did capture the intensity in the year to year variability in the present biennial epidemic patterns, however, the actual RVGE cases in even year rotavirus season were much lower than the value predicted by our model.

**Reviewer #2:** The authors focus on an important aspect of mechanisms which may influence the effectiveness of vaccines in general and in the particular setting of RV vaccination. To estimate the reduction of RVGE incidence by efforts to improve vaccination rates by FP by applying a deterministic age-structured dynamic model is a feasible approach. Overall, this is a well-written manuscript with sound results.
However, I have some comments to deal with:
I doubt whether the biennial pattern of RVGE in U.S. may be explained only by incomplete vaccine coverage. Authors should provide more evidence for this hypothesis by literature or own experience. Trend for biennal patterns or oscillations in RVGE associated hospitalizations was seen by others (e.g. Prelog M et al., J Inf Dis 2016). Authors should comment on this. The immunological or virological mechanisms behind biennial patterns or disappearance of biennial patterns should be explained to understand the association with vaccine coverage.
Assumptions in table 1 are mainly based on few articles. Authors should comment why they took this choice and did not perform a systematic review approach previous to the modeling procedure. Authors should state why the articles by Velazquez or Heymann are prior to others if there are any on these parameters.

**Response**: Thank you for your comments and suggestions

**Response to biennial patterns**:

In general, biennial patterns of RVGE could be caused by(a combination of) vaccine coverage, vaccine efficacy, and birth rate (Pitzer 2009; Pitzer 2011). Since birth rates in the U.S have been low and fairly stable through the pre and post – vaccine era, and previous studies have indicated that incomplete vaccine coverage can drive biennial patterns, we considered vaccine coverage as the main factor in our model driving biennial patterns in the U.S. Note that these patterns do not occur in most other high-income countries with rotavirus immunization. We incorporated a detailed explanation of this rationale in the text (lines 83-89). Additionally, we also mentioned the disappearance of biennial patterns due to increasing of vaccine coverage predicted in the Weidemann 2014 modeling study in the text (lines 288 – 289)

**Edits to text (lines 83 – 89)**:

In general, biennial patterns of disease could be caused by a combination of vaccine coverage rates, vaccine efficacy, and birth rate, such that susceptibles are accumulated at a certain rate (Pitzer 2009; Pitzer 2011, Shah 2018). In one study, a RVGE transmission model predicted that biennial patterns of RVGE after introduction of vaccine when birth rates are lower while an annual pattern of RVGE was predicted when birth rates are high (Pitzer 2011). In the U.S, birth rates have remained fairly stable0 in the pre and post-vaccine era. Thus, birth rate is likely not a significant driver of the biennial pattern of RVGE in the U.S.

**Response to parameters selection**:

We used data from the Velazquez 1996 study to inform the proportions of infection, symptomatic infection and severe disease and relative infectiousness of non-primary infections because of its rigorous study design and study population. Velazquez 1996 was a birth cohort study conducted in Mexico quantifying risk reduction after infection by collecting stool weekly from 200 infants from birth to 2-year-old. There are three birth cohort studies conducted in Mexican, Indian and African birth cohorts that quantify the reduced risk of subsequent infections. As there are no published birth cohort studies conducted in the U.S., we selected the Velazquez study of the Mexican birth cohort which would have more similar demographic characteristics to the U.S. than the birth cohorts from India and Africa. Moreover, the data from the Velazquez study has been used to parameterize many other rotavirus transmission models (Pitzer 2012; Atchison 2010; Atkins 2012).

The work by Prelog et al (Jid 2018) is an important paper showing the range of indirect effects of rotavirus immunization in Australia, but does not provide parameter input for our model.

The Hymann 2015 study provides data on the natural history of rotavirus such as daily rate of loss of maternal immunity and daily rate of loss infection. These data have been used to inform parameters in several transmission model (Atchison 2010, Atkins 2012).
